# Directed ortho,ortho'-dimetalation of hydrobenzoin: Rapid access to hydrobenzoin derivatives useful for asymmetric synthesis

**DOI:** 10.3762/bjoc.7.154

**Published:** 2011-09-22

**Authors:** Inhee Cho, Labros Meimetis, Lee Belding, Michael J Katz, Travis Dudding, Robert Britton

**Affiliations:** 1 Department of Chemistry, Simon Fraser University, 8888 University Drive, Burnaby, B.C., Canada, V5S 1S6; 2 Department of Chemistry, Brock University, 500 Glenridge Ave, St Catharines, ON, Canada, L2S 3A1

**Keywords:** chiral diol, directed ortho-metalation, hydrobenzoin

## Abstract

A variety of ortho,ortho'-disubstituted hydrobenzoin derivatives are readily accessible through a directed ortho,ortho'-dimetalation strategy in which the alcohol functions in hydrobenzoin are deprotonated by *n*-BuLi and the resulting lithium benzyl alkoxides serve as directed metalation groups. The optimization and scope of this reaction are discussed, and the utility of this process is demonstrated in the one-pot preparation of a number of chiral diols as well as a short synthesis of the chiral ligand Vivol.

## Introduction

**Figure 1:**
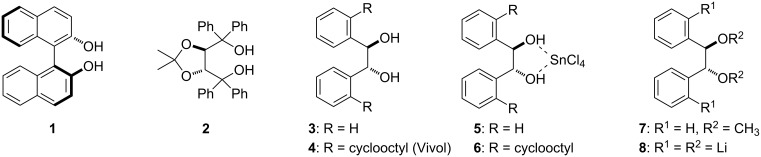
Chiral diols useful for asymmetric synthesis and the tetralithio intermediate **8**.

The discovery of new chiral ligands and auxiliaries continues to expand the frontiers of catalytic asymmetric synthesis. In particular, *C*_2_-symmetric diols, such as (*S*)-BINOL (**1**) [1] and (−)-TADDOL (**2**) [2] (Figure 1), have garnered considerable attention owing to the wide variety of asymmetric reactions promoted by these ligands and/or their derivatives. Although hydrobenzoin (e.g., **3**) has not been utilized to the same extent, it has also demonstrated utility as both a chiral ligand [3–8] and auxiliary [9–17]. For example, Hall reported that the hydrobenzoin·SnCl_4_ complex **5** promotes the allylboration of hydrocinnamaldehyde with modest enantioselectivity (26% ee) [3], and a hydrobenzoin–ytterbium complex was found to catalyze asymmetric aldol/Evans–Tishchenko reactions [5]. Moreover, the hydrobenzoin dimethyl ether **7** was shown to direct the asymmetric addition of organolithium reagents to arene tricarbonylchromium complexes [7] and α,β-unsaturated aldimines [8]. Notably, derivatives of hydrobenzoin in which the aromatic rings have been functionalized in the ortho and ortho' positions often display improved diastereo- or enantioselectivity over the parent diol **3** [3,14]. For example, the addition of cyclooctyl rings to the ortho and ortho' positions of the hydrobenzoin·SnCl_4_ complex (i.e., Vivol·SnCl_4_ (**6**)) leads to a dramatic improvement in enantioselectivity in the allylboration of hydrocinnamaldehyde (93% ee) [3]. Unfortunately, while (*R*,*R*)- and (*S*,*S*)-hydrobenzoin are relatively inexpensive [18], or can be readily prepared on kilogram-scale from *trans-*stilbene through Sharpless asymmetric dihydroxylation (SAD) [19–20], the synthesis of ortho,ortho'-functionalized derivatives of hydrobenzoin typically requires several steps that include McMurry coupling of an ortho-substituted benzaldehyde followed by I_2_-catalyzed isomerization of the resulting stilbene and subsequent SAD [21–22]. Thus, while various hydrobenzoin derivatives have been reported, their multi-step synthesis, the modest enantioselectivity in the SAD step [22], the problems associated with their optical enrichment [6], and the necessary determination of optical purity for each derivative, all complicate the rapid preparation of congeneric libraries. To address these issues, we recently reported a new process for the direct functionalization of (*R*,*R*)-hydrobenzoin (**3**) [23] through a directed ortho,ortho'-dimetalation strategy in which the alcohol functions in hydrobenzoin are deprotonated by *n*-BuLi and the resulting lithium benzyl alkoxides serve as directed metalation groups (DMGs) [24–28] and facilitate formation of the tetralithio intermediate **8**. Herein, we provide a detailed account of this work as well as the application of these methods to a short synthesis of the chiral diol Vivol (**4**).

## Results and Discussion

**Scheme 1:**
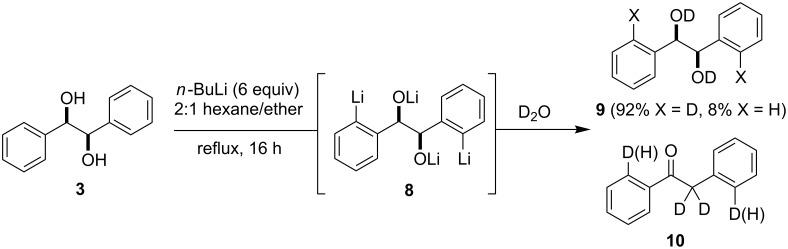
Directed ortho,ortho'-dimetalation of (*R*,*R*)-hydrobenzoin (**3**).

As indicated in Scheme 1, the optimized conditions for the directed ortho,ortho'-dimetalation of (*R*,*R*)-hydrobenzoin (**3**) involve the treatment of a solution of **3** under reflux with an excess (6 equiv) of *n*-BuLi, followed after 16 h by treatment with an electrophile [23]. The optimization of this process relied on a series of D_2_O quenching studies and analysis of the ^1^H NMR and mass spectra derived from the crude reaction products. These studies led to the eventual selection of a 2:1 mixture of hexane/ether as the most favorable reaction solvent for the formation of the tetralithio intermediate **8** [23].

Despite the high level of deuterium incorporation (92%) observed in the reaction of the tetralithio intermediate **8** with D_2_O (Scheme 1), this result was not observed for reactions with other electrophiles (see below). In addition, the products from deuterium quenching studies were often accompanied by the formation of varying amounts of the deoxybenzoin **10**, which presumably derives from reaction of an alkoxide function in **8** with D_2_O, followed by deuteroxide elimination/enolate formation and subsequent reactions with D_2_O. Bearing this in mind, the directed ortho,ortho'-dimetalation of hydrobenzoin was reinvestigated using I_2_ as the electrophile quench. As indicated in Table 1, the use of stoichiometric (i.e., 4 equiv, entry 1) amounts of *n*-BuLi led to low isolated yields of the diiodohydrobenzoin **12**. Successively increasing the equivalents of *n*-BuLi resulted in approximate increases of 15% in the isolated yield of **12**, with a maximum yield of roughly 50% achieved when 6 equiv of *n*-BuLi were employed (entries 2 and 4). Although the use of 8 equiv of *n*-BuLi led to a slightly higher isolated yield of **12** (53%), and only 8 hours were required for the formation of the tetralithio intermediate (entries 6 and 7), the conditions identified in entry 4 require fewer equivalents of base and electrophile and were consequently selected as the optimal reaction conditions [23]. Notably, addition of TMEDA (entries 3 and 5), which would presumably assist in the disaggregation of organolithium species, failed to improve these results and in fact led to lower conversion and isolated yields of the diiodohydrobenzoin **12**.

**Table 1:** Optimization of the synthesis of the diiodohydrobenzoin **12**.

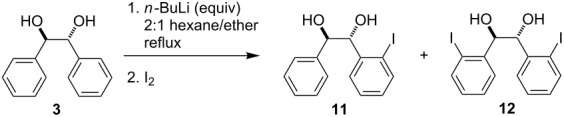					

entry	*n-*BuLi [equiv]	reflux time [h]	I_2_ [equiv]	ratio **11**:**12**^a^	isolated yield **12** [%]

1	4	16	5	1:1	20
2	5	16	6	1:1.7	34
3	5^b^	16	6	2.6:1^c^	8
4	6	16	7	1:5	51
5	6^d^	16	7	2:1^e^	13
6	8	16	9	1:5	53
7	8	8	12	N.D.^f^	51

^a^Ratio determined by analysis of ^1^H NMR spectra recorded on crude reaction mixture. ^b^TMEDA (5 equiv) was added prior to the addition of *n*-BuLi. ^c^Hydrobenzoin (48%) was also recovered from this reaction. ^d^TMEDA (6 equiv) was added prior to the addition of *n*-BuLi. ^e^Hydrobenzoin (34%) was also recovered from this reaction. ^f^Not determined.

**Figure 2:**
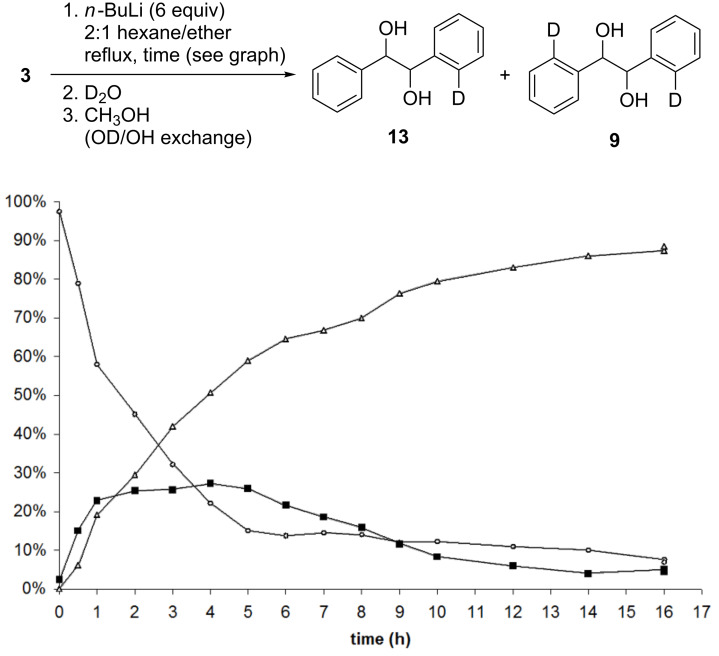
Percentage of (*R*,*R*)-hydrobenzoin (**3**) (○), monodeuterohydrobenzoin (**13**) (■), and dideuterohydrobenzoin (**9**) (Δ) as determined by mass spectrometry (ESI) [23].

During the evaluation of the reaction of the tetralithio intermediate **8** with I_2_ (Table 1), we were surprised to find that monoiodohydrobenzoin **11** was produced in equivalent or lower yield than the diiodohydrobenzoin **12**, even at low overall conversion (e.g., Table 1, entries 1 and 2). These results suggested that the rate-limiting step in the formation of the tetralithio intermediate **8** may be the first DoM (i.e., formation of a trilithio intermediate) and that the second DoM event is relatively more rapid. To gain further insight into this process, the reaction of (*R*,*R*)-hydrobenzoin (**3**) with *n*-BuLi followed by treatment with D_2_O [23] or CH_3_I was monitored by mass spectrometry and ^1^H NMR spectroscopy, respectively. As indicated in Figure 2, the results of the D_2_O quenching study were in accordance with our original observation: A relatively slow removal of the first ortho proton is followed by a second, more rapid DoM event.

**Figure 3:**
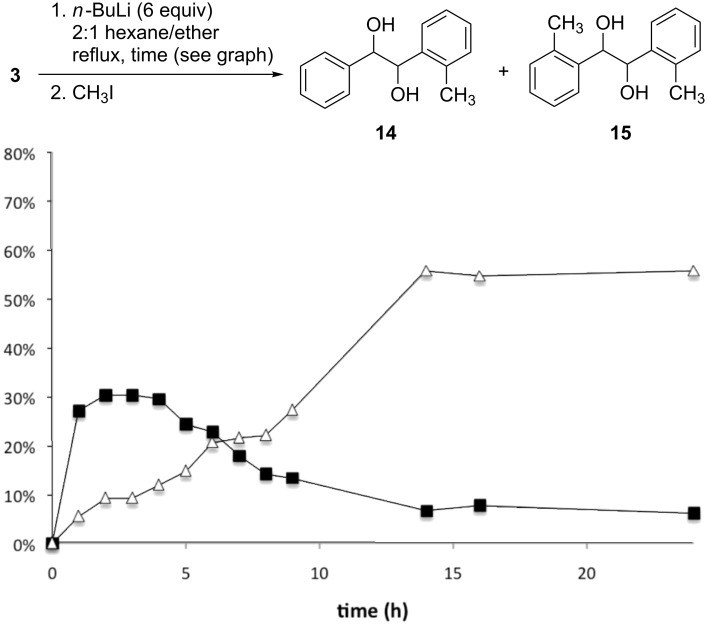
Percentage of methylhydrobenzoin (**14**) (■), and dimethylhydrobenzoin (**15**) (Δ) as determined by ^1^H NMR spectroscopy. The remainder of the material is recovered (*R*,*R*)-hydrobenzoin (**3**).

When the progress of the sequential ortho-metalations was monitored by quenching with CH_3_I, however, the difference in rates was not as pronounced, and only in the case where the formation of the tetralithio intermediate **8** was allowed 6 hours at reflux was the proportion of mono- and dimethylhydrobenzoin (i.e., **14** and **15**) equivalent. It is notable that the differing results depicted in Figure 2 and Figure 3 may be attributed to intra- or intermolecular deprotonation of the *o*-tolyl group following reaction of the tetralithio intermediate **8** with one equiv of CH_3_I, leading to the formation of misleading amounts of the monomethylhydrobenzoin **14**, or simply differing reactivity of the tetralithio intermediate with D_2_O and CH_3_I.

**Scheme 2:**
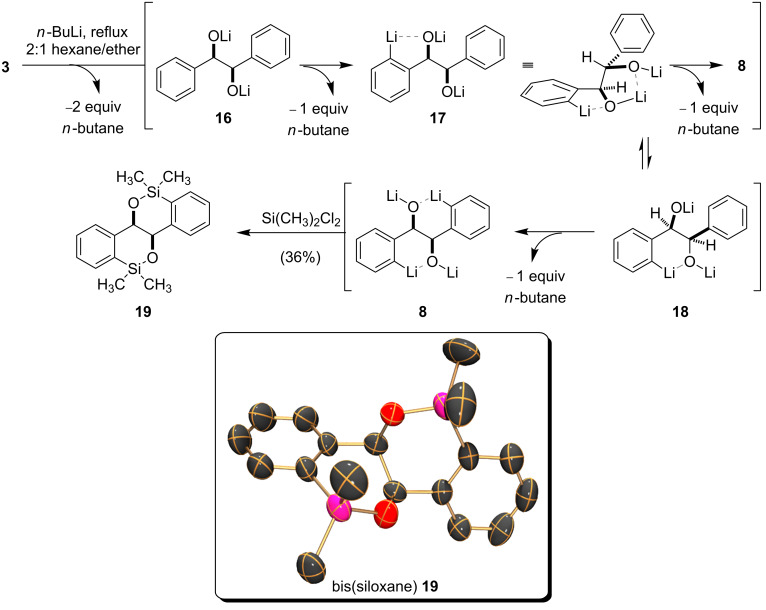
Formation of the tetralithio intermediate **8** and the X-ray crystal structure of the bis(siloxane) **19**.

A possible explanation for the differing rates of DoM events observed during the D_2_O quenching studies is depicted in Scheme 2 [29]. Thus, following the first ortho-metalation, the intermediate aryl lithium **17** (presumably in an aggregated form) may adopt a conformation in which the lithium alkoxide (DMG) and consequently the base (i.e., *n*-BuLi or its aggregates) are positioned in close proximity to the ortho proton that is to be removed. Alternatively, the intermediate aryl lithium **17** may undergo rearrangement to the six-membered heterocycle **18**, in which the cis-relationship between the distal lithium alkoxide and the unlithiated phenyl ring facilitates the second deprotonation. While the latter proposal is less likely, based on Seebach’s observation that 2-phenylethanol does not undergo ortho-lithiation [30], reaction of the tetralithio intermediate **8** with dimethyldichlorosilane led to the formation of the bis(siloxane) **19** (not the 5-membered ring bis(siloxane) isomer), whose structure was unambiguously confirmed by X-ray crystallographic analysis (see Scheme 2, inset).

**Scheme 3:**
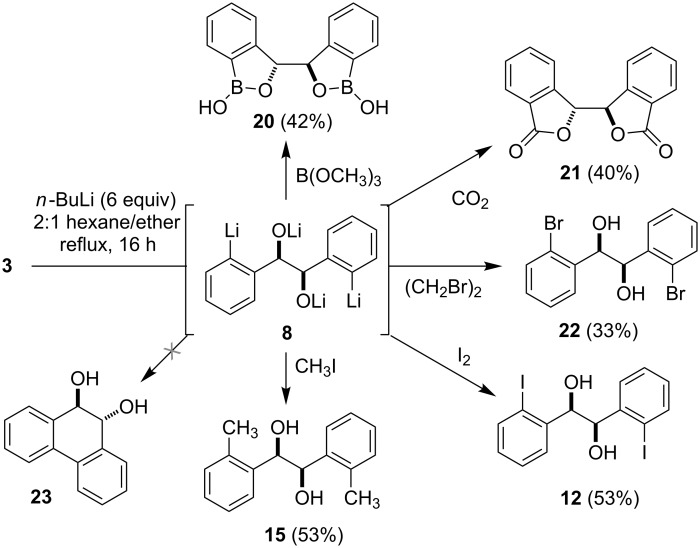
Reaction of the tetralithio intermediate **8** with various electrophiles.

As indicated in Scheme 3, formation of the tetralithio intermediate **8** and subsequent reaction with various electrophiles led to synthetically useful yields of ortho-functionalized hydrobenzoin derivatives [23]. In each case, approximately 5–10% of the corresponding monofunctionalized hydrobenzoin (e.g., **14**, Figure 3) was also produced, but was readily separable from the desired product by flash chromatography. Unfortunately, reaction of the tetralithio intermediate **8** with carbonyl electrophiles (e.g., acetone, acetaldehyde, diethyl carbonate, valeraldehyde, cyclohexanal, crotonaldehyde, formaldehyde, DMF, benzaldehyde), trimethylsilyl chloride, or allyl/benzyl chloride/bromide failed to provide the desired products in reasonable yield. For example, treatment of the tetralithio intermediate **8** with benzyl chloride resulted in recovery of (*R*,*R*)-hydrobenzoin along with 1-chloro-1,2-diphenylethane, the latter of which arises through the deprotonation of benzyl chloride by **8** and reaction of the resulting benzyl anion with a second equivalent of benzyl chloride. Likewise, attempts to effect a reductive coupling of the tetralithio intermediate to afford the dihydrophenanthrenediol **23** [22] with the aid of various copper or iron halides [31], or palladium(II)chloride [32] were unsuccessful. In an effort to fine-tune the procedure by attenuating the reactivity of the tetralithio intermediate **8**, transmetalation with ZnCl_2_ or MgBr_2_ followed by treatment with various electrophiles was also investigated. Disappointingly, these efforts failed to offer any improvement in the coupling of **8** with DMF, benzaldehyde, or allyl bromide and consequently we focused our attention on reactions of the readily available diiodohydrobenzoin **12** and bis(benzoxaborol) **20**.

**Scheme 4:**
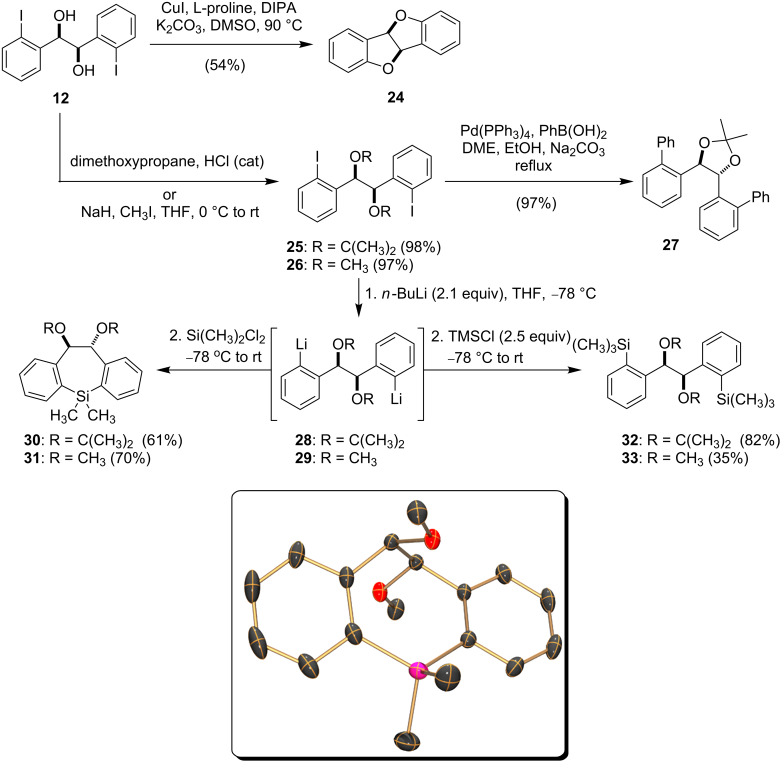
Reactions of the diiodohydrobenzoin **12** and X-ray crystal structure of the dihydrosilepin **31**.

As highlighted in Scheme 4, attempts to directly engage diiodohydrobenzoin **12** in Cu-catalyzed C–N cross-coupling reactions [33] led only to the formation of *cis*-4b,9b-dihydrobenzofuro[3,2-*b*]benzofuran (**24**) [34]. Consequently, the diol **12** was converted to the corresponding acetonide **25** or methyl ether **26** prior to cross-coupling. In this manner, the diphenylhydrobenzoin derivative **27** could be accessed in excellent overall yield [3,23]. Alternatively, lithium–halogen exchange carried out on diiodohydrobenzoins **25** or **26**, followed by reaction with various electrophiles affords access to a wider array of hydrobenzoin derivatives than those highlighted above in Scheme 3 [17]. For example, electrophiles such as TMSCl and dichlorodimethylsilane engage in high yielding reactions with the dianions **28** and **29** to provide the silyl-functionalized hydrobenzoin derivatives **30**–**33**.

**Scheme 5:**
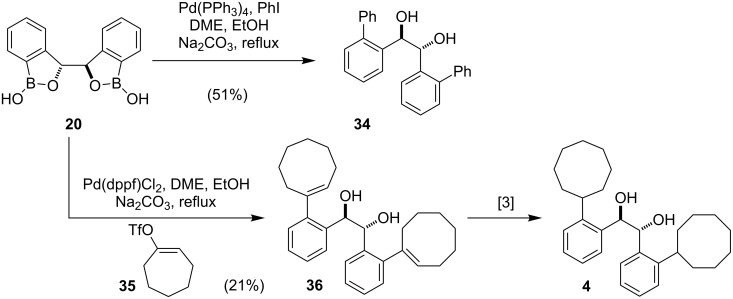
Cross coupling reactions of the bis(benzoxaborol) **20** and a short formal synthesis of (*R*,*R*)-Vivol (**4**).

The bis(benzoxaborol) **20** also proved to be a versatile intermediate for the synthesis of hydrobenzoin derivatives through Pd-catalyzed cross-coupling reactions (Scheme 5). This approach compliments those described above and avoids the need to protect the diol function prior to the cross-coupling step. Notably, purification of the bis(benzoxaborol) **20** proved unnecessary as the diphenylhydrobenzoin **34** was prepared in an overall yield of 32% from (*R*,*R*)-hydrobenzoin (**3**) without purification of **20**, compared to 21% when the intermediate bis(benzoxaborol) **20** was purified by column chromatography [23]. Finally, the efficiency of this process was demonstrated in a short formal synthesis of (*R*,*R*)-Vivol (**4**). Thus, a Pd-catalyzed cross-coupling of the known triflate **35** [35] with the bis(benzoxaborol) **20** afforded the dicyclooctenylhydrobenzoin **36**, which was reduced in quantitative yield to afford Vivol (**4**) by Hall [3]. Notably, this three-step procedure for the preparation of optically pure Vivol compares well with the reported synthesis and should be effective for the rapid production of new ligands for asymmetric synthesis.

## Conclusion

In conclusion, an efficient and economical process was developed for the direct functionalization of hydrobenzoin that relies on a directed ortho,ortho'-dimetalation strategy. Importantly, a wide variety of chiral diols (e.g., Vivol (**4**)) are now readily accessible in optically pure form following this one-pot reaction. Although the range of electrophiles that engage in synthetically useful reactions with the tetralithio intermediate **8** is limited, the diiodohydrobenzoin **12** and bis(benzoxaborol) **20** are both prepared in good yield and are readily derivatized through cross-coupling reactions. We are currently exploring the utility of the bis(benzoxaborol) **20** and derivatives of this substance as chiral Lewis acids and will report our findings in due course.

## Supporting Information

File 1 Experimental details and characterization data for all compounds.

File 2 X-ray crystallographic information files (CIFs) for crystals of **19** and **31**.
