# Supplementary material for: Directed ortho,ortho'-dimetalation of hydrobenzoin: Rapid access to hydrobenzoin derivatives useful for asymmetric synthesis
Source: Beilstein J Org Chem. 2011 Sep 22;7:1315–22. doi: 10.3762/bjoc.7.154 (PMC3201044; doi:10.3762/bjoc.7.154)
Supplement: File 1 — Experimental details and characterization data for all compounds. [file Beilstein_J_Org_Chem-07-1315-s001.pdf]

## Supporting Information

## for

# Directed ortho,ortho'-dimetalation of hydrobenzoin: Rapid access to hydrobenzoin derivatives useful for asymmetric synthesis

Inhee Cho[1], Labros Meimetis[1], Lee Belding[1,2], Michael J. Katz[1], Travis Dudding[2], Robert Britton[1,]*

Address: [1]Department of Chemistry, Simon Fraser University, 8888 University Drive, Burnaby, B.C., Canada, V5S 1S6 and [2]Department of Chemistry, Brock University, 500 Glenridge Ave, St Catharines, ON, Canada, L2S 3A1

Email: Robert Britton - rbritton@sfu.ca

## Experimental details and characterization data for all compounds.

## Experimental

**General remarks:** All reactions described were performed under an atmosphere of dry argon in oven-dried or flame-dried glassware. THF, $Et_2O$, hexanes and toluene were used directly from an MBraun Solvent Purifier System (MB-SP Series) or purchased in Dri-solv bottles (EMD). Flash chromatography was carried out with 230–400 mesh silica gel (E. Merck, Silica Gel 60) following the technique described by Still [1]. Concentration of solvents was accomplished with a Büchi rotary evaporator with an acetone/dry-ice condenser, and removal of trace solvent was accomplished with a Welch vacuum pump. NMR spectra were recorded using deuteriochloroform ($CDCl_3$) as the solvent. Signal positions are given in parts per million (ppm) from tetramethylsilane (δ 0) and were measured relative to the signal of the solvent ($CDCl_3$: δ 7.26, $^1$H NMR; δ 77.16, $^{13}$C NMR) [2]. Coupling constants (*J* values) are given in Hertz (Hz) and are reported to the nearest 0.1 Hz. $^1$H NMR spectral data are tabulated in the order: Multiplicity (s, singlet; d, doublet; t, triplet; m, multiplet), number of protons, coupling constant(s). Proton nuclear magnetic resonance ($^1$H NMR) and carbon nuclear magnetic resonance ($^{13}$C NMR) spectra were recorded on a Bruker 600 ($^1$H, 600 MHz; $^{13}$C, 150 MHz), Varian Inova 500 ($^1$H, 500 MHz; $^{13}$C, 125 MHz), or Varian Inova 400 ($^1$H, 400 MHz; $^{13}$C, 100 MHz) as indicated. Infrared (IR) spectra were recorded on MB-series Bomem/Hartman & Braun Fourier transform spectrophotometer with internal calibration as KBr pellets or as thin film on NaCl plates. Only selected, characteristic absorption data are provided for each compound. *n*-Butyllithium was purchased from Aldrich chemical company and titrated using menthol in THF containing 2,2'-bipyridyl as an indicator [3]. Mass

spectrometry was recorded by the Simon Fraser University Mass Spectrometry Services Laboratory using either electron impact (EI) or electrospray (ESI). Optical rotation was measured on a Perkin Elmer Polarimeter 341.

**X-ray crystallography**

Suitable crystals of **19** and **31** for single crystal X-ray diffraction were mounted on glass fibers by epoxy adhesives. Data for both crystals was collected on a Bruker Smart system equipped with an APEX II CCD detector and graphite-monochromated molybdenum fine-focused X-ray tube operated at 50 kV and 30 mA (1.5 kW). The data was collected and processed using the APEX II software suit. Structures were solved using direct methods (SIR 92) and refined through Fourier techniques in CRYSTALS. Both crystals grow in non-centrosymmetric spacegroups. The presence of silicon atoms in the structure was sufficient to accurately determine the handedness of the crystal from anomalous dispersion with a molybdenum source (i.e., the flack parameter was found to be 0.02(8) and 0.02(8) for **19** and **31**, respectively). The structure of **31** consists of one independent molecule per asymmetric unit while the structure of **19** consists of 3 molecules per asymmetric unit. Further crystallographic details can be found in the .cif file (Supporting Information File 2).

**General Procedure (formation of the tetralithio intermediate 8)**

To a stirred suspension of (*R*,*R*)-(+)-hydrobenzoin (**3**) in a mixture of hexane and ether was added *n*-BuLi (6 equiv) dropwise at rt (the total ratio of hexane:ether was 2:1 and the

[**3**] was 0.1 M). The yellow reaction mixture was then heated at reflux for 16 h resulting in the formation of a deep orange/red solution [4].

**(4b*R*,9b*R*)-cis-4b,9b-Dihydrobenzofuro[3,2-*b*]benzofuran (24)**

A stirred mixture of (*1R*,*2R*)-1,2-bis(2-iodophenyl)-ethane-1,2-diol (**12**) (100 mg, 0.22 mmol), CuI (8 mg, 0.04 mmol), L-proline (10 mg, 0.08 mmol), diisopropylamine (0.09 mL, 0.66 mmol), and $K_2CO_3$ (61 mg, 0.44 mmol) in DMSO (2.0 mL) was heated to 90 ºC and maintained at this temperature for 12 h. After this time, the reaction mixture was concentrated and the crude product was purified by flash column chromatography (10:1 hexane/EtOAc) to afford *cis*-4b,9b-dihydrobenzofuro[3,2-*b*]benzofuran (**24**) (25 mg, 54%) as a colorless solid (mp: 154 °C); $[\alpha]_D^{25}$ +371 (*c* 0.50, $CHCl_3$); $^1$H NMR (500 MHz, $CDCl_3$) δ 7.55 (d, 2H, *J* = 7.5 Hz), 7.29 (t, 2H, *J* = 8.0 Hz), 6.98 (t, 2H, *J* = 7.5 Hz), 6.89 (d, 2H, *J* = 8.0 Hz), 6.29 (s, 2H); $^{13}$C NMR (125 MHz, $CDCl_3$) δ 160.1, 131.5, 126.7, 124.5, 121.3, 111.0, 86.6; IR (thin film): 3050, 2923, 1601, 1481, 1463, 958, 746 cm $^{-1}$; HRMS (*m/z*): $[M + H]^+$ calcd for $C_{14}H_{11}O_2$, 211.0759; found, 211.0755.

**(1*R*,2*R*)-1,2-Dimethoxy-1,2-bis(2-iodophenyl)ethane (26)**

To a cold (0 ºC), stirred solution of (1*R*,2*R*)-1,2-bis(2-iodophenyl)ethane-1,2-diol (**12**) (2.00 g, 4.29 mmol) in THF (43 mL) was added NaH (60% dispersion in mineral oil, 360 mg, 9.0 mmol). After 5 min, iodomethane (0.8 mL, 12.9 mmol) was added in one portion and the reaction mixture was allowed to warm to rt and stirred for 12 h. After this time, the reaction mixture was treated with $H_2O$ (10 mL) and $Et_2O$ (20 mL) and the layers were separated. The aqueous layer was washed with $Et_2O$ (2 x 20 mL) and the combined

organic extracts were dried ($MgSO_4$), filtered and concentrated to provide (1*R*,2*R*)-1,2-dimethoxy-1,2-bis(2-iodophenyl)ethane (**26**) as a colorless solid (2.11 g, 97%) (mp: 99–102 °C); $[\alpha]_D^{25}$ −75.4 (*c* 0.71); IR (thin film): 2931, 1462, 1084, 1008, 743 cm $^{-1}$; $^1$H NMR (400 MHz, $CDCl_3$) δ 7.65 (dd, 2H, *J* = 8.0, 1.2 Hz), 7.63 (dd, 2H, *J* = 7.9, 1.7 Hz), 7.34 (dt, 2H, *J* = 7.7, 1.2 Hz), 6.92 (dt, 2H, *J* = 7.8, 1.7 Hz), 4.81 (s, 2H), 3.22 (s, 6H); $^{13}$C NMR (100 MHz, $CDCl_3$) δ 139.9, 139.2, 130.6, 129.8, 128.2, 100.8, 88.6, 56.9; HRMS (*m/z*): $[M]^+$ calcd for $C_{16}H_{16}O_2I_2$, 493.9240; found, 493.9233.

**Dihydrosilepin 31**

To a cold (−78 ºC), stirred solution of (1*R*,2*R*)-1,2-dimethoxy-1,2-bis(2-iodophenyl)ethane (**26**) (200 mg, 0.40 mmol) in $Et_2O$ (8.0 mL) was added *n*-BuLi (0.34 mL, 0.85 mmol, 2.5 M in hexanes) dropwise. The resulting slightly yellow solution was stirred for 10 min and then dichlorodimethylsilane (58 µL, 0.48 mmol) was added dropwise. After additional 3 h at −78 ºC, the reaction mixture was treated with a saturated aqueous solution of $NH_4Cl$ and extracted with $Et_2O$ (3 x 15 mL). The combined organic layers were washed with brine, dried ($MgSO_4$), and concentrated. Purification of the crude product by flash column chromatography (20:1 hexane/EtOAc) afforded the tricyclic silane **31** as colorless crystals (84 mg, 70%) (mp: 95–98 ºC); $[\alpha]_D^{25}$ −29.2 *c* 0.82, $CHCl_3$); IR (thin film): 2894, 1246, 1092, 944, 815, 735 cm$^{-1}$; $^1$H NMR (400 MHz, $CDCl_3$) δ: 7.71–7.73 (m, 2H), 7.29–7.38 (m, 6H), 4.84 (s, 2H), 3.13 (s, 6H), 0.49 (s, 6H); $^{13}$C NMR (100 MHz, $CDCl_3$) δ: 143.2, 138.6, 134.9, 131.3, 128.5, 127.1, 86.4, 56.7, 0.7; HRMS (*m/z*): $[M]^+$ calcd for $C_{18}H_{22}O_2Si$, 298.1389; found: 298.1388.

**(1*R*,2*R*)-1,2-Dimethoxy-1,2-bis(2-trimethylsilylphenyl)ethane (33)**

To a cold (−78 ºC), stirred solution of (1*R*,2*R*)-1,2-dimethoxy-1,2-bis(2-iodophenyl)ethane (**26**) (200 mg, 0.40 mmol) in $Et_2O$ (4.0 mL) was added *n*-BuLi (0.45 mL, 0.85 mmol, 1.87 M in hexanes) dropwise. The resulting slightly yellow solution was stirred for 30 min and then chlorotrimethylsilane (130 µL, 1.00 mmol) was added dropwise. After an additional 3 h at −78 ºC, the reaction mixture was treated with a saturated aqueous solution of $NH_4Cl$ and extracted with $Et_2O$ (3 x 15 mL). The combined organic layers were washed with brine, dried ($MgSO_4$), and concentrated. Purification of the crude product by flash column chromatography (10:1 hexane/EtOAc) afforded (1*R*,2*R*)-1,2-dimethoxy-1,2-bis(2-trimethylsilylphenyl)ethane (**33**) as colorless crystals (54 mg, 35%) (mp: 34–37 ºC); $[\alpha]_D^{25}$ −73.7 (*c* 0.41, $CHCl_3$); IR (thin film): 2926, 1246, 1107, 836, 738 $cm^{-1}$; $^1H$ NMR (400 MHz, $CDCl_3$) δ: 7.51 (dd, 2H, $J$ = 7.5, 1.2 Hz), 7.21 (dt, 2H, $J$ = 7.3, 1.4 Hz), 7.12 (dt, 2H, $J$ = 7.7, 1.5 Hz), 6.81 (dd, 2H, $J$ = 7.8, 0.9 Hz), 4.87 (s, 2H), 3.18 (s, 6H), 0.31 (s, 18H); $^{13}C$ NMR (100 MHz, $CDCl_3$) δ: 143.5, 140.3, 134.5, 128.6, 127.9, 126.7, 84.4, 56.3, 1.8; HRMS (*m/z*): $[M]^+$ calcd for $C_{22}H_{34}O_2Si_2$, 386.2097; found: 386.2099.

**(1*R*,2*R*)-1,2-Bis(2-cyclooctenylphenyl)ethane-1,2-diol (36)**

To a stirred solution of the vinyl triflate **35** [5] (150 mg, 0.58 mmol) and Pd(dppf)$Cl_2$ (10.6 mg, 0.013 mmol) in dimethoxyethane (1.5 mL), was added a solution of the bis(benzoxaborol) **20** (35 mg, 0.13 mmol) dissolved in a minimal amount of EtOH (0.2 mL), followed by a solution of $Na_2CO_3$ (0.3 mL, 0.6 mmol, 2 M in $H_2O$). The resulting mixture was heated at reflux for 24 h, then cooled to rt and filtered through

celite. The filtrate was concentrated to provide a residue, which was diluted with $Et_2O$ and washed with brine, dried ($MgSO_4$), filtered and concentrated. Purification of the crude product by flash column chromatography (7:3 hexane/EtOAc) afforded (1*R*,2*R*)-1,2-bis(2-cyclooctenyl-phenyl)ethane-1,2-diol (**36**) (12 mg, 21%). The spectral data derived from **36** was consistent with that reported by Hall [6].

## References


1. Still, W.C.; Kahn, M.; Mitra, A. *J .Org. Chem.* **1978,** *43*, 2923–2925.
2. Gottlieb, H.E.; Kotlyar, V.; Nudelman, A. *J. Org. Chem.* **1997,** *62*, 7512–7515.
3. Lin, H; Paquette, L.A. *Synth. Commun.* **1994,** *24*, 2503–2506.
4. For experimental details regarding the reaction of the tetralithio intermediate **8** with electrophiles discussed in this manuscript see: Cho, I.; Meimetis, L.; Britton, R. *Org. Lett.* **2009**, *11*, 1903–1906.
5. Scheiper, B.; Bonnekessel, M.; Krause, H.; Furstner, A. *J. Org. Chem.* **2004**, *69*, 3943–3949.
6. Rauniyar, V.; Zhai, H.; Hall, D. G. *J. Am. Chem. Soc.* **2008**, *130*, 8481–8490.
